# Mechanisms of Feedback Regulation of Vitamin A Metabolism

**DOI:** 10.3390/nu14061312

**Published:** 2022-03-21

**Authors:** Catherine O’Connor, Parisa Varshosaz, Alexander R. Moise

**Affiliations:** 1 MD Program, Northern Ontario School of Medicine, 317-MSE Bldg., 935 Ramsey Lake Rd., Sudbury, ON P3E 2C6, Canada; caoconnor@nosm.ca; 2 Biology and Biomolecular Sciences Ph.D. Program, Northern Ontario School of Medicine, Laurentian University, Sudbury, ON P3E 2C6, Canada; pvarshosaz@laurentian.ca; 3 Medical Sciences Division, Northern Ontario School of Medicine, 317-MSE Bldg., 935 Ramsey Lake Rd., Sudbury, ON P3E 2C6, Canada; 4 Department of Chemistry and Biochemistry, Biology and Biomolecular Sciences Program, Laurentian University, Sudbury, ON P3E 2C6, Canada

**Keywords:** carotenoids, homeostasis, retinoids, retinoic acid receptor, metabolism, negative feedback, nuclear hormone receptor, transcriptional regulation

## Abstract

Vitamin A is an essential nutrient required throughout life. Through its various metabolites, vitamin A sustains fetal development, immunity, vision, and the maintenance, regulation, and repair of adult tissues. Abnormal tissue levels of the vitamin A metabolite, retinoic acid, can result in detrimental effects which can include congenital defects, immune deficiencies, proliferative defects, and toxicity. For this reason, intricate feedback mechanisms have evolved to allow tissues to generate appropriate levels of active retinoid metabolites despite variations in the level and format, or in the absorption and conversion efficiency of dietary vitamin A precursors. Here, we review basic mechanisms that govern vitamin A signaling and metabolism, and we focus on retinoic acid-controlled feedback mechanisms that contribute to vitamin A homeostasis. Several approaches to investigate mechanistic details of the vitamin A homeostatic regulation using genomic, gene editing, and chromatin capture technologies are also discussed.

## 1. Introduction

Intercellular signaling relies on hormones, cytokines, neurotransmitters, autacoids and other signaling mediators which activate specific receptor proteins. Depending on the location of their receptor, binding of a ligand to its receptor can occur on the cell surface or inside the cell. While surface receptors activate more rapid responses involved in sensory, immune, and neuronal signaling cascades, intracellular receptors mediate transcriptional changes that allow the cell to adapt to extracellular and environmental inputs by changing its metabolism, fate, or differentiation. Hydrophilic signaling molecules (peptides, amines) associate with receptors localized at the cell surface, consisting of ligand-gated channels, receptor tyrosine kinases, or G protein-coupled receptors (GPCRs) which trigger a wide plethora of intracellular signaling activities. Meanwhile, lipophilic hormones (retinoids, sterols and other lipid signaling mediators) cross the target cell’s membrane and bind to intracellular receptors which carry out transcriptional regulation. There are, however, exceptions with many examples of lipophilic signaling molecules (eicosanoids, sphingosine 1-phosphate) which interact primarily with surface receptors, as well as examples of lipophilic signaling mediators that carry out signaling activities via both surface and intracellular receptors [1].

Nuclear hormone receptors (NHRs) represent a family of ligand-dependent transcription factors which share an evolutionarily conserved modular domain architecture (reviewed in [2,3]. The N-terminal (A/B) domain is variable and disordered and includes a region that interacts with various coregulators. This is followed by the DNA-binding (C) domain which contains two zinc-finger motifs which bind specific response elements (RE) found in enhancer regions that controls target genes. A flexible hinge domain (D) separates the DNA-binding domain from the C-terminal ligand-binding domain (E), which as its name implies confers ligand selectivity. A second cofactor interacting region (AF-2) is located within the ligand-binding domain. NHRs can function as monomers, homodimers, or heterodimers. Binding of ligand to the ligand-binding domain (E) results in a conformational change which is allosterically transmitted to the DNA-binding, cofactor recruitment regions, and can also be imparted to domains residing with the dimeric partner.

NHRs can be classified based on their signaling mechanism [3]. Unliganded type I receptors such as estrogen and progesterone receptors are found within the cytoplasm in association with chaperone proteins. Upon binding ligand, type I receptors translocate to the nucleus where they associate with inverted repeat DNA motifs as homodimers. Type II receptors include thyroid hormone and retinoic acid receptors, which form heterodimers with the retinoid X receptor (RXR) and are found located in the nucleus bound to DNA, and are associated and with co-repressor and histone deacetylases (HDACs) complexes in the absence of ligand. Binding of ligand allows type II NHR to disassociate from co-repressor complexes and bind to co-activators, which allows for transcription of target genes. Type III and IV receptors have similar mechanism as type I NHRs but differ in terms of their dimerization and type of DNA response elements which they recognize. A unified nomenclature system categorizes NHR members based on phylogenetically related families [4].

NHR ligands are involved in both short-range and long-range signaling. Some NHR ligands such as steroids, thyroid hormones, 1α,25-dihydroxyvitamin D_3_, can travel far from their source via circulation to reach target organs and carry out endocrine signaling. For other NHR ligands the main circulating form in serum is an inactive precursor—such NHRs are primarily involved in short-range paracrine signaling to the same cell where the ligand is produced (autocrine), or to neighboring cells (paracrine). Circulating forms of various lipophilic hormones or prohormones are associated with either specific or non-specific serum binding proteins. These carrier proteins include those involved in the transport of thyroid hormones (transthyretin, thyroxine-binding globulin), retinol (retinol binding protein 4), steroid hormones (corticosteroid binding globulin, sex hormone-binding globulin), and other sterols (vitamin D binding protein). In addition, lipophilic hormones and precursors can also associate with lipoproteins or with non-specific serum proteins (serum albumin, alpha-fetoprotein).

Both excess and deficiency of an NHR ligand can lead to disease through alterations in the signaling pattern of the respective NHR. To maintain the appropriate level of the active form of the signaling mediator, a biological system needs to actively adjust the rate of synthesis, secretion, transport, and breakdown of a hormone. The capacity to maintain internal normalcy despite changes in the external environment is a cardinal feature of all endocrine regulators and ensures the homeostasis of a biological system [5]. Nuclear hormone homeostasis relies largely on negative feedback regulation. In general, perturbations in hormone levels trigger adaptive changes in the expression of genes coding for transporters, binding proteins, and synthetic and catabolic enzymes involved in hormone metabolism. Often transcription of genes involved in hormone metabolism is regulated directly or indirectly via the same NHR as the one targeted by the specific hormone. In the case of NHR ligands derived from dietary precursors, as in case of vitamin A, feedback regulation also needs to account for changes in the chemical nature, level, or absorption of dietary precursors. We will focus here on the adaptive changes that adjust the production of the active forms of vitamin A in response to environmental factors such as stressors, changes in diet, and interference with vitamin A metabolism by drugs, toxins, and disease.

## 2. Bioactive Vitamin A Metabolites

Vitamin A is a key nutrient in the human diet and is especially important to sustain vision, embryonic development, immunity, and tissue repair and homeostasis. Dietary compounds with vitamin A activity encompass both preformed all-*trans*-retinol (referred to here as retinol for simplicity), and retinyl esters, as well as provitamin A carotenoids, such as β-carotene or β-cryptoxanthin. The intake of vitamin A from either preformed vitamin A or provitamin A carotenoids is reported as retinol activity equivalent (RAE) which is equal to 1 µg of retinol, 12 µg of β-carotene, or 24 µg of α-carotene or β-cryptoxanthin [6]. Provitamin A carotenoids can be recognized based on having at least one unmodified ionone ring. Yet, in some species, several naturally occurring compounds with modified β-ionone rings can also meet some vitamin A-specific visual functions, for example, vitamin A2 (all-*trans*-3,4-didehydroretinol) which is derived from all-*trans*-retinol and used as a visual chromophore by freshwater fish and amphibians, and vitamin A3 (all-*trans*-3-hydroxy-retinal) which is derived from xanthophyll carotenoids and is used as a visual chromophore by insects. Synthetic and natural chemical species that carry out vitamin A biological activities are known as retinoids [7]. Many synthetic retinoids are clinically employed in the treatment of skin disorders and cancers. In nature, all vitamin A compounds are derived through the biotransformation of carotenoids synthesized by various photosynthetic and non-photosynthetic organisms including plants, fungi, and bacteria. Both preformed vitamin A and provitamin A carotenoid precursors represent important sources of vitamin A in the human diet [6].

The best understood bioactive forms of vitamin A are 11-*cis*-retinaldehyde and all-*trans*-retinoic acid. Within the visual process, the photosensitive chromophore 11-*cis*-retinaldehyde is covalently coupled to GPCRs of the opsin family (represented by melanopsin and cone and rod opsins). Every photon of light isomerizes 11-*cis*-retinaldehyde to all-*trans*-retinaldehyde which is recycled back to 11-*cis*-retinaldehyde via the visual cycle [8]. The non-visual functions of vitamin A are accomplished via all-*trans*-retinoic (RA), a ligand of the retinoic acid receptors (RAR)-α, -β, and -γ (classified NR1B1-B3, respectively) [9]. RAR isoforms form heterodimers with the retinoid X receptors (RXR)-α, -β, and -γ (NR2B1-B3, respectively) resulting in nine possible RAR-RXR combinations, not considering additional isoforms derived through alternate splicing. The RA isomer, 9-*cis*-RA, is a potent ligand of both RAR and RXR and can activate RXR homodimers and permissive RXR heterodimers in certain settings [10,11,12,13,14,15]. In addition to RA and 11-*cis*-retinal, several other vitamin A metabolites have also been shown to exhibit biological activities. *Retro*-retinoids and ring-oxidized forms of retinol and RA, such as 14-hydroxy-4,14-retro-retinol, anhydroretinol, 4-oxo-retinoids can be detected in tissues and have been shown to carry out signaling activities in some settings [16,17,18,19,20,21,22,23,24,25].

Retinoids containing one saturated double bond, *dihydro*-retinoids, represent an emerging class of potential bioactive vitamin A metabolites. Some *dihydro*-retinoids, are derived enzymatically via retinol saturase (RETSAT), which stereospecifically converts all-*trans*-retinol to (13*R*)-all-*trans*-13,14-dihydroretinol, which then acts as a precursor to (13*R*)-all-*trans*-13,14-dihydroretinoic acid [26,27,28]. Zebrafish RETSAT can also catalyze the formation of all-*trans*-7,8-dihydroretinol [29]. All-*trans*-13,14-dihydroretinoic acid is a selective and potent RAR ligand in vitro, but the levels and transcriptional activity of all-*trans*-13,14-dihydroretinoic acid in vivo are much lower than those seen for RA [27,30,31]. In addition, several *cis*-*dihydro*-retinoid metabolites, such as 9-*cis*-13,14-dihydroretinoic acid and its 4-oxo-metabolite can be detected in vivo in significant quantities and were suggested to act as endogenous ligands of RXR [32,33,34,35,36,37]. However, genetic evidence for a role of *dihydro*-retinoids in the activation of RXR or other NHRs is still lacking [30,38]. For instance, *Retsat*-deficiency affects a multitude of biological processes involving lipid metabolism, immune response, and oxidative stress, yet, none of these effects appear to be mediated by its currently known all-*trans*-13,14-dihydroretinol product [31,39,40,41,42,43].

In addition to its canonical transcriptional activities via RAR-RXR, RA can also carry out alternate modes of signaling (reviewed in [44]). RA-RAR can result in non-genomic effects through activation of kinase signaling pathways such as p38 mitogen-activated protein (MAP) kinase pathway and the PI3 kinase pathway [45,46,47]. Other non-genomic activities of RA and retinol have also been implicated in regulation of metabolism, cell growth and synaptic plasticity [48,49,50,51,52].

In conclusion, accumulating evidence suggests that vitamin A can operate via alternate metabolites other than 11-*cis*-retinaldehyde and RA. Evidence also suggests that both known and novel bioactive retinoid metabolites can signal via alternate pathways outside those involved in vision or in transcriptional regulation via RAR/RXR. However, our understanding of these alternate functions of vitamin A is limited and more support is needed to appreciate the biological relevance of such effects [53,54].

## 3. Transcriptional Regulation Mediated by RAR-RXR

RA plays important roles in embryonic development and adult life (reviewed in [55]. RA is required during embryogenesis for anterior-posterior patterning and organogenesis [55,56,57,58]. For this reason, even modest changes in the levels of RA in embryonic tissues can lead to developmental defects and embryonic lethality [56,59,60]. In postnatal life, RA is required to sustain the function and regeneration of tissues [61]. Changes in tissue RA levels in adults are associated with impaired immunity and reproduction, and cardiovascular, skin, and metabolic disorders [62,63,64,65,66].

Activation of RAR-RXR leads to extensive changes in the transcriptional landscape and protein composition of cells [67]. Treatment of cultured cells with RA leads to upregulation or downregulation in the expression of thousands of transcripts (referred to as differentially expressed genes or DEGs). A significant portion of transcript changes are also mirrored in changes in the levels of corresponding proteins [60]. In addition, studies based on chromatin immunoprecipitation-high-throughput sequencing (ChIP-Seq) using antibodies directed against RAR reveal that the number of DNA sequences occupied by RAR is, in fact, much greater in number than the number of DEGs [68,69]. The number of genes confirmed to be regulated by RA in vivo is also considerable. Such changes in gene expression can be seen in animals exposed to RA excess, or to inhibitors of RA formation [60]. For example, transcriptome analysis showed that the expression of thousands of genes is altered in embryos as a result of ablation of the gene coding for the RA synthetic enzyme RALDH2 [70]. We will briefly review the mechanism of transcriptional activation by RAR-RXR and we refer the reader to several recent reviews for more details regarding this topic [55,71].

**Figure 1:**
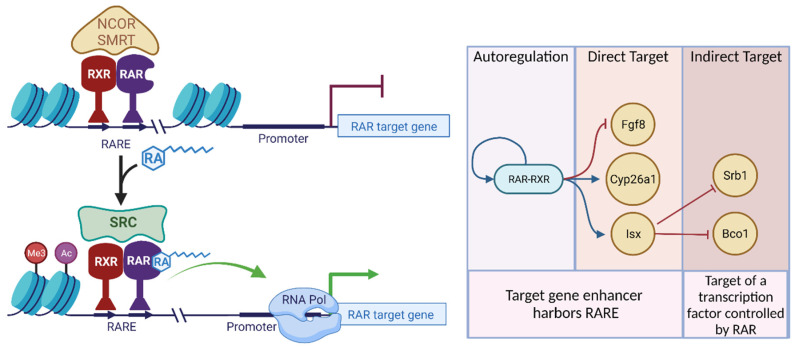
Transcriptional mechanism of RAR-RXR. RAR-RXR binds RARE element found in enhancer regions of target genes. Binding of RA to RAR-RXR causes the dissociation of co-repressors such as NCOR/SMRT and associated HDAC enzymes, and the recruitment of co-activators such as SRC which include HAT and histone methylase enzymes. Conversely, binding RA allows for ligand-induced repression of certain targets by RAR. Chromatin opening allows the initiation of transcription by RNA polymerases. RAR-RXR can control the expression of its own encoding genes (Rara, Rarb, Rarg) or direct target genes that harbor a RARE. Many retinoid-responsive genes are also controlled indirectly via intermediary RA-responsive transcription factors. Created with BioRender.com (accessed on 16 February 2022).

Transcriptional regulation by RA via its cognate receptors RAR-RXR operates in a similar manner as other type II NHR and is outlined in Figure 1. Briefly, the DNA binding or C-domain of the RAR-RXR complex typically recognize a specific response element (RARE) which consists of direct repeats (DRs) of the RGKTCA motif separated by a spacer of one, two or five nucleotides, and which are referred to as DR1, DR2, or DR5, respectively [72]. Despite the acceptance and use of canonical DR motifs to predict NR-binding sites, NRs often recognize DNA sequences in a promiscuous manner, including variations in the orientation and sequence of the hexameric motif, DRs with different spacer length (DR0) and in some cases even half sites [73,74]. RAREs are found within *cis*-acting regulatory domains of genes such as enhancers, which can be found upstream, or downstream, within introns and often at a considerable distance from target genes. An enhancer harboring a RARE can act bidirectionally to increase transcription of a target promoter on the same chromosome, and there are examples where the same RARE can serve multiple genes [75].

The activity of RAR-RXR is regulated by the RA ligand. In the absence of ligand, RAR-RXR is associated with a co-repressor complex composed of Silencing Mediator of Retinoic acid and Thyroid hormone receptor (SMRT)/Nuclear Receptor Corepressor (NCo-R) and HDACs [76,77,78]. RA binding to the F, or ligand binding domain of RAR leads to a conformational change which allows RAR-RXR to recruit coactivators protein complexes such as SRC-1 (NCOA1) and histone acetylases (HAT) which mediate chromatin relaxation and enhance promoter activity [79]. Different cell types express a different repertoire of RAR-RXR co-regulators which impart a cell type-specific context for RAR-RXR activity. In addition to ligand-dependent transactivation, ligand-bound RAR-RXR can also induce repression through recruitment of specific repressive complexes to the enhancer domains of specific gene targets [80,81] reviewed in [82]. In addition, at any given time, the number of gene regulatory elements occupied by RAR is much greater than the number of genes whose expression can be altered by RA treatment, which suggests that there are secondary, post-receptor mechanisms which control the activity of RA-bound RAR. Though, the three isoforms of RAR exhibit non-overlapping gene target and tissue expression patterns, deficiency of only one RAR isoform (*Rara^−/−^*, *Rarb^−/−^*, and *Rarg^−/−^*) can be compensated to a large extent by remaining isoforms. However, deficiency of more than one isoform of RAR as seen in combination knockout mice *Rara^−/−^Rarb^−/−^*, *Rara^−/−^Rarg^−/−^*, *Rarb^−/−^Rarg^−/−^* or *Rara^−/−^Rarb^+/−^Rarg^−/−^* results in lethality [83,84]. Many of the defects observed in RAR combination mutants recapitulate those seen in severely RA deficient mice.

## 4. Vitamin A Supplementation

Vitamin A deficiency is a significant public health concern which, despite large scale supplementation campaigns, affects the lives of millions of children and women of childbearing age in developing countries [85]. At the same time, mitigation of vitamin A deficiency based on supplementation of large doses of preformed vitamin A (60,000 mcg RAE (200,000 IU) can lead to hypervitaminosis A which leads to bone resorption and impaired growth in children, and to hip fractures and osteoporosis in older adults [86,87]. Even a moderately increased intake of preformed vitamin A (vitamin A from supplements > 10,000 IU/day) can be associated with increased incidence of birth defects related to impaired development of neural crest derived structures (neurocristopathies) [88]. This increased incidence is particularly concerning given that current tolerable upper intake levels for pregnant women are 9333–10,000 IU/day (recommended dietary allowance, RDA for pregnant women is 2500–2567 IU retinol/day).

Even as β-carotene is a much safer form of vitamin A supplementation compared to preformed retinol from the point of view teratogenicity, high β-carotene intake can negatively interact with environmental stressors and comorbidities to result in an increased risk of disease [89,90]. On the other hand, all retinoid-based therapies are known to carry a high risk of toxicity and teratogenicity. These studies and clinical observations, argue that an effective and safe retinoid therapy and vitamin A supplementation program should ensure proper vitamin A-supported functions, but do so in a manner that safeguards against the deleterious effects of retinoid excess. A better understanding of the regulatory feedback processes that govern the metabolism of vitamin A is important for the development of safer supplementation programs.

The pathways responsible for vitamin A uptake and delivery, and for the synthesis and breakdown of RA have been the subject of several excellent recent reviews [55,91,92,93,94,95]. In the current review, we will focus primarily on the mechanisms through which RA controls its own metabolism.

## 5. Vitamin A Absorption

**Figure 2:**
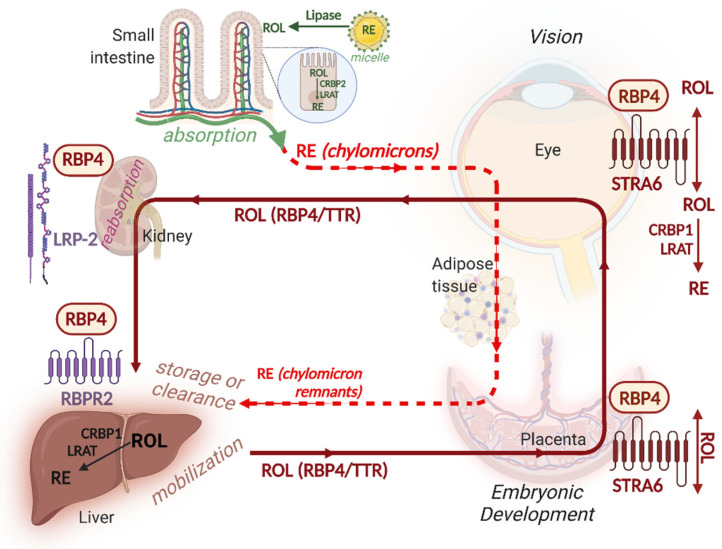
The absorption and delivery of vitamin A. Vitamin A is absorbed from the lumen of the small intestine following hydrolysis of retinyl esters (RE) and re-esterification of retinol via cellular retinol binding protein 2 (CRBP2) and lecithin:retinol acyltransferase (LRAT). REs are secreted by enterocytes as part of chylomicrons and circulate via the lymphatic system (green) and enter the circulation (dashed red). Chylomicrons are hydrolyzed via lipoprotein lipase (LPL) to deliver retinol to target tissues such as the eye, adipose tissue and placenta and return to be cleared by the liver as remnants. Liver stores retinol as RE in HSC through the action of LRAT. When needed hepatic stellate cells (HSC) hydrolyze RE and secrete retinol bound to retinol binding protein 4 (RBP4) in association with transthyretin (TTR) in the circulation. RBP4 can both deliver as well as take up retinol from tissues that express its receptor stimulated by retinoic acid 6 (STRA6). RBP4 is reabsorbed from the proximal tubule of the kidney via lipoprotein receptor-related protein 2 (LRP-2 or megalin)–cubilin complex. A hepatic RBP4 receptor RBPR2 may also play a role in the uptake of RBP4 by the liver. During fasting the liver can also secrete RE in conjunction with VLDL as an alternate means to mobilize retinol (not shown). Created with BioRender.com (accessed on 16 February 2022).

Uptake of vitamin A from the intestinal lumen conforms to the general mechanism of lipid absorption and is outlined in Figure 2. Bile salts solubilize lipids and aid incorporation of retinyl esters and carotenoids into mixed micelles which pass through the unstirred layer to reach the intestinal brush border membrane. Diseases associated with impaired bile synthesis or secretion can lead to vitamin A deficiency [96]. Pancreatic lipases hydrolyze retinyl esters to retinol. Bile acid synthesis and secretion are both increased to promote vitamin A uptake in mice maintained on a vitamin A deficient (VAD) diet—conversely in vitamin A sufficiency bile acid synthesis is reduced [97]. 

Uptake of retinol from the intestinal lumen does not appear to require a specific receptor but it does exploit mass action kinetics through the esterification of retinol within brush border cells. The most important enzyme involved in retinyl ester synthesis in the intestine as well as other tissues is lecithin:retinol acyltransferase (LRAT) which transfers fatty acids obtained from the *sn*-1 position of various phospholipids to retinol [98,99]. Several other enzymes with acyl-CoA dependent transferase (ARAT) activity have also been shown to carry out the esterification of retinol, however, but their activity appears to play a role primarily in mammary glands and skin [100]. Genetic ablation studies of putative ARAT enzymes have failed to show profound or specific effects on retinol esterification [101,102,103,104]. In addition to LRAT, enterocytes also express cellular retinol binding protein 2 (CRBP2 encoded by *RBP2*) which is required for delivery of retinol to LRAT for esterification (reviewed in [94,105]). Maternal *Rbp2* loss-of-function in mice results in fetal mortality when dams are fed diets containing more moderate vitamin A levels (4 IU of retinyl palmitate/g) [106]. Retinyl esters synthesized in enterocytes are packaged in chylomicrons assembled through the activity of microsomal triglyceride transfer protein (MTP) and secreted into the lymphatic circulation [104]. In peripheral tissues, chylomicrons undergo remodeling and retinyl esters are hydrolyzed by lipoprotein lipase (LPL) and taken up by target organs, such as eye, adipose tissue. A majority of retinyl esters remain associated with chylomicron remnants and are cleared by the liver.

Esterification of retinol within enterocytes is responsive to vitamin A status. The expression of *Rbp2* and *Lrat* are both induced by RA. Together these activities sequester retinol and retinyl esters and reduce synthesis of RA [94]. Though, no RAREs have so far been conclusively demonstrated within the promoter of *Lrat* or *Rbp2*, several DNA regions responsible for the induction of *Lrat* by RA have been identified [107]. Induction of LRAT and CRBP2 by RA most likely occurs indirectly [107,108,109]. Inclusion of RA in vitamin A supplementation approaches (VA combined with retinoic acid (VARA) elegantly exploits the induction of LRAT by RA to increase the intestinal absorption and retention of retinol as retinyl esters in extrahepatic tissues [110,111].

Carotenoid uptake is facilitated by several transporters shared with other lipid-soluble vitamins and sterols such as scavenger receptor class B type 1 (SR-B1, encoded by *Scarb1*) and CD36 (refs. [112,113,114,115,116,117,118,119] reviewed in [95]). A large fraction of β-carotene is cleaved within brush border cells through the activity of beta-carotene-dioxygenase 1 (BCO1) to afford retinaldehyde which is then reduced to retinol via the action of retinal reductases enzymes which are members of the microsomal short-chain dehydrogenase reductase (SDR) family. Β-carotene-derived retinol can be esterified via LRAT and secreted by enterocytes in conjunction with chylomicrons. Provitamin A carotenoids that retain only one unmodified β-ionone group can also be converted to retinol via beta-carotene-dioxygenase 2 (BCO2) to produce apo-10′-carotenals, which are subsequently converted to retinaldehyde by BCO1 [120,121,122,123,124]. The fraction of β-carotene which remains uncleaved in enterocytes is incorporated into nascent chylomicrons which are secreted into the lymphatic circulation to reach peripheral organs and can be cleared by the liver as remnants. Carotenoids can also be found in association with other lipoprotein fractions (apoB100 and HDL) through hepatic secretion and/or exchange [125,126].

The absorption of carotenoids is influenced by genetic polymorphisms that affect genes involved in β-carotene uptake and by the variable nature of the food matrix components [127]. These factors result in large variations in an individual’s ability to absorb and convert provitamin A carotenoids to vitamin A. Despite these variations, the level of circulating serum retinol is relatively stable. This homeostatic effect is even more evident in the case of diets relying largely on provitamin A carotenoids to meet vitamin A needs. One factor that contributes to the capacity to control the uptake and conversion of provitamin A carotenoids has to do with a negative regulatory pathway which operates in the intestinal epithelium via the intestine-specific homeobox transcription factor (ISX) [95].

Approximately 70% of the β-carotene absorbed by brush border cells is cleaved via BCO1 to retinaldehyde which contributes to the intracellular pool of retinol. Retinol is oxidized via yet-to-be identified SDR enzymes to retinaldehyde, and subsequently oxidized by retinaldehyde reductases (RALDH encoded by *Aldh1a*) enzymes to produce RA. Within the enterocyte RA activates RAR-RXR to induce the expression of ISX via an RARE located within its promoter [128,129]. ISX represses the expression of both *Srb1* and *Bco1* and therefore restricts the uptake and conversion of β-carotene [115,128,130,131]. Since the levels of RA within the enterocyte are proportional to the levels of available retinol, the ISX-mediated feedback mechanism prevents formation of unnecessary retinol in states of vitamin A sufficiency. As a result, *Isx*-deficient mice have no ability to control β-carotene uptake and conversion [115,128,130,131]. A similar feedback mechanism operates at the fetal-maternal interface where high dietary retinol restricts β-carotene uptake by fetal tissues [125]. The amount of retinol available for RA synthesis within enterocytes is controlled by LRAT. Ablation of *Lrat* in mice leads to exaggerated feedback due to high levels of available retinol [132]. It is important to note, that a negative feedback mechanism does not appear to operate in the case of the intestinal uptake of dietary preformed vitamin A (retinol, retinyl esters) which are incidentally are associated with a much higher risk of teratogenicity and toxicity compared to provitamin A carotenoids.

## 6. Vitamin A Storage

Retinyl esters associated with chylomicrons are taken up by the parenchymal liver cells and hydrolyzed to retinol which becomes associated with a cellular retinol binding protein 1 (CRBP1) [133,134,135,136]. CRBP1 plays important roles in fine-tuning vitamin A metabolism (reviewed in [94]). First, CRBP1 protects retinol from degradation and spurious reactions and ensures delivery of retinol to retinoid enzymes for oxidation or esterification. Secondly, there is evidence that CRBP1 controls the rate of retinyl ester utilization. A high ratio of apo- to holo-CRBP1 acts to inhibit LRAT and stimulate retinyl ester hydrolase activity. Conversely holo-RBP1 induces esterification and oxidation of retinol (reviewed in [94]). *Crbp2* loss-of-function in mice only produced obvious phenotypes of retinol deficiency when mice are not provided a vitamin A sufficient diet [106], whereas *Crbp1* knockout mice are normal and viable [137], but have reduced capacity to synthesize RA [51,138].

Hepatocytes transfer retinol to a specialized cell population called hepatic stellate cells (HSC) [133]. In HSCs, retinol is esterified via LRAT to produce retinyl esters which are incorporated in lipid droplets [139]. The mechanism of retinol transfer from hepatocytes to HSCs is not clear. Similar retinyl esters storage particles as found in HSCs are also seen in retinal pigmented epithelium (RPE), lung cells and pancreatic stellate cells [140,141,142,143]. Adipose tissue, lung, kidney, and RPE also store a fraction of vitamin A. There is also evidence for β-carotene being stored in the liver in HSCs, and that the converting enzyme BCO1 is expressed in both HSCs and parenchymal hepatic cells [144,145,146]. Therefore, provitamin A precursors represent another potential hepatic storage mechanism for vitamin A.

Hepatic retinol stores can be mobilized upon increased demand. As needed, retinyl esters of HSCs are hydrolyzed via several hepatic lipases and transferred to hepatocytes [147,148,149,150,151,152]. Hepatocytes secrete retinol bound to retinol binding protein (RBP, encoded by RBP4), and associated with transthyretin (TTR) [153,154,155,156,157]. There is evidence that retinyl esters can form in adipose tissue independently of LRAT, perhaps via an ARAT enzyme, and that these stores can also be mobilized in times of deficiency [158]. Similarly, RBP4 can also be expressed in other tissues such as adipose tissue, but RBP4-derived from non-hepatic sites does not play a significant role in systemic vitamin A metabolism [159,160]. However, ectopic overexpression of RBP4 in muscle tissues can rescue the delivery of vitamin A to eye tissues when endogenous RBP4 expression is lacking [161,162].

Storage of retinol is under strict feedback regulation by RA. Expression of liver *Lrat* and *Rbp1* is induced by RA, thus acting to direct retinol flux toward storage in times of vitamin A sufficiency [108,163]. Not surprisingly, vitamin A metabolism is also responsive to regulators of liver lipid metabolism. Mechanistically, farnesoid X receptor (FXR) was shown to influence LRAT expression and the levels of hepatic retinyl esters [96,164]. Meanwhile, RAR/RXR signaling promotes the expression of apolipoprotein C-III, represses the expression of synthetic enzyme CYP7A1, and influences the expression of various bile acid transporters [97,165,166] reviewed in [167]. The ramifications of the reciprocal influence of bile and vitamin A metabolism are also relevant for understanding the role of vitamin A in the pathological mechanisms of liver disease such as NAFLD and steatohepatitis. Retinoid stores disappear as HSC become activated during liver disease. Activated HSC also contribute to liver pathology by transdifferentiating to myofibroblasts [168,169]. However, despite the correlation of HSC activation and loss of HSC retinyl esters stores, the causal relationship between the two events is not clear (reviewed in [170]).

Even though the visual system recycles spent chromophore, it still requires a constant supply of retinol precursor to maintain vision; if not, lack of supply of retinol can lead to night blindness. Though, stimulated by retinoic acid 6 (STRA6, see below) and LRAT both respond to RA, it is not clear if these genes are RA responsive in RPE cells. The expression of visual cycle enzymes including LRAT, BCO1, RDH10, RDH11 and RPE65 increases with age. There is also evidence that *Lrat* expression in the RPE is driven by retinoid by-products of the visual cycle (A2E and all-*trans*-retinal) which activate RAR most likely via conversion to RA [171]. The activity of LRAT in the eye is not only required for storing vitamin A but also to form the precursor for the enzyme RPE65, the isomerohydrolase that regenerates 11-*cis*-retinaldehyde. The induction of *Lrat* by RAR via agonistic activity of RA derived from visual cycle byproducts is not surprising, but this positive feedback could be detrimental considering the pathology of age-related macular degeneration. An overactive visual cycle can lead to accumulation of cytotoxic visual cycle metabolites and result in photoreceptor death [172].

## 7. Vitamin A Delivery to Target Tissues

Retinol-bound RBP4 interacts with specific receptors expressed by target tissues. STRA6 is a high affinity holo-RBP4 receptor expressed by many blood—tissue barrier sites such as retinal pigmented cells, placenta, yolk sac, choroid plexus, and Sertoli cells [173,174]. Interaction of RBP4 with STRA6 allows for the bidirectional transfer of retinol into and out of cells [174,175,176,177,178,179]. Liver and intestine cells do not express *Stra6*, but express another RBP4 receptor (RBPR2) [180]. RBPR2 is proposed to allow for the return excess of retinol via RBP4 to the liver for storage or clearance. Genetic studies suggest that RPBR2 is also required for photoreceptor morphogenesis in zebrafish [181]. A mouse deficient in *Rbpr2* (also known as Stra6-like, *Stra6l*) has increased corneal opacity and hematopoietic defects [182]. Interestingly, the primate homologue of RBPR2 is encoded by two separate genes which translate into two separate proteins with correspond to the N- and C-terminal domains of mouse RBPR2 [180]. It remains to be determined if primate RBPR2 proteins function as receptors for RBP4.

The TTR-holoRBP4 complex is composed of a TTR tetramer and RBP4 found in 1:1 stoichiometry in circulation where RBP4 levels are limiting [183]. The TTR-RBP4 complex is larger than the glomerular filtration cutoff, however, in the absence of TTR, the 21 kDa RBP4 protein is easily filtered. As a result, TTR-deficiency results in a drastic reduction (from 6 h to 0.5 h) in the half-life of RBP4 in serum [184]. A similar effect is induced by fenretinide (N-(4-hydroxyphenyl) retinamide) and other agents which disrupts the association of TTR and RBP4 [185]. Even under normal circumstances a small fraction of RBP4 becomes free of TTR and is filtered by the kidney. There is evidence that filtered RBP4 can be reabsorbed from the proximal tubule via endocytosis carried out by low density lipoprotein receptor-related protein 2 (LRP-2, megalin)–cubilin complex [186,187,188].

In addition to protein-mediated transport, a considerable fraction of vitamin A can be transported by lipoproteins which deliver retinoids to many target tissues including the placenta. The importance of lipoprotein-mediated RE transport is evident in both patients and mice deficient in RBP4 (refs. [189,190,191] reviewed in [192]). LPL controls the binding and hydrolysis of apo-CII bearing lipoproteins in peripheral tissues. These fractions include intestinal-derived chylomicrons postprandially, and hepatic-derived VLDL during fasting. Maternal–fetal transport of retinoids relies on RBP4 (both maternal and fetal-derived) as well as lipoprotein-mediated pathways, both of which are responsive to vitamin A status (refs. [193,194,195] reviewed in [196]).

The transport and delivery of vitamin A to target tissues is controlled by feedback regulation. Both *Rbp4* expression and RBP4 protein secretion respond to vitamin A status [155,197,198,199,200,201]. Meanwhile, *Stra6* is induced by RA and is a direct target of RAR [173,174,202,203]. Recent structural and biochemical evidence suggests that the intracellular domain of STRA6 associates with the calcium-binding protein calmodulin. This association is proposed to allow intracellular calcium to control the direction of retinol transfer via STRA6 [204]. It is not clear whether regulation of STRA6 by calcium is part of a feedback mechanism that controls the uptake or export of retinol in response to the retinoid needs of the target cells. In contrast to *Stra6*, expression of *Rbpr2* is negatively correlated with levels of hepatic retinoids, serum retinol and holo-RBP4 and RA [180]. The expression of *Lrp-2* is itself also induced by RA [205].

## 8. Conversion of Retinol to RA

**Figure 3:**
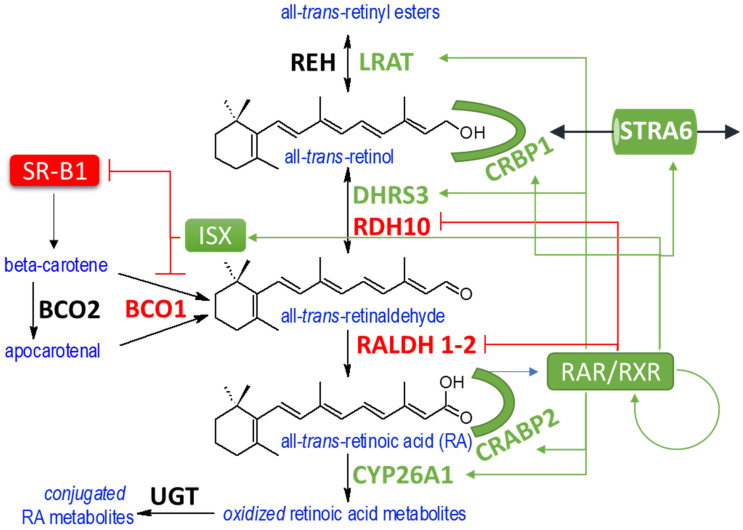
Factors involved in the feedback regulation in metabolism of RA. The influence of RA on genes involved in the pathway of conversion of vitamin A precursors to RA is shown only for retinoid genes currently known to respond to RA. Other enzymes, transporters and binding proteins involved in retinoid metabolism are not shown but are listed in text and enclosed references. Names of enzymes, transporters and binding proteins involved in retinoid metabolism are listed in red if downregulated by RA, or green if upregulated by RA, and in black if regulation by RA is currently not known.

Retinol is converted to RA via sequential oxidations as depicted in Figure 3. Retinol is oxidized to retinaldehyde by microsomal enzymes which belong to the SDR family, and which couple retinol oxidation or retinaldehyde reduction, with the reduction of NAD or oxidation of NADPH cofactor, respectively. The SDR family is one of the largest known enzyme families and its members are involved in the transformation of a wide range of substrates including various lipids, eicosanoids and steroids.

Interconversion of retinol and retinaldehyde is a critical step in the formation of RA and in the formation and recycling of 11-*cis*-retinaldehyde, as part of the visual cycle. As a result, there has been considerable effort made to identify the enzymes responsible for this important retinoid biotransformation. Biochemical approaches, involving heterologous expression and retinoid oxidation/reduction assays using candidate enzymes, have implicated a significant number of SDR enzymes in the oxidation/reduction of retinol and retinal, respectively (refs. [206,207,208,209,210,211] reviewed in [61,92]). However, genetic loss-of-function studies support a role in the retinaldehyde–retinol interconversion for a more limited number of SDRs (reviewed in [92]). Other enzymes with retinaldehyde reductase activity include several aldo-keto reductase (AKR) enzymes and cytosolic alcohol dehydrogenases (ADHs) belonging to the medium-chain alcohol dehydrogenase family, but their contribution to vitamin A metabolism under physiological conditions is still not clear [212,213].

Loss-of-function approaches have led to the identification of the two primary SDRs responsible for the interconversion retinol to retinaldehyde during embryonic development. Retinol dehydrogenase 10 (RDH10) is an NAD-dependent retinol oxidase whose deletion results in embryonic lethality and a deficiency of RA [214]. Conversely, dehydrogenase/reductase (SDR family) member 3 (DHRS3) carries out the reduction of retinaldehyde to retinol using NADPH [59,215]. *Dhrs3*-ablation also results in embryonic lethality but in this case the lethality results from excess RA. DHRS3 and RDH10 carry out opposite activities in the conversion of retinol to retinaldehyde based on the different reduced/oxidized ratio of their preferred dinucleotide cofactor. The developmental consequences of *Rdh10* and *Dhrs3* ablation involve skeletal, and cardiovascular defects [60,214,216,217,218,219,220,221]. We refer the reader to Shannon et al. for a summary of the developmental consequences of *Rdh10-* and *Dhrs3*-ablation in mice [56]. The embryonic lethality caused by *Rdh10*- and *Dhrs3*-deletion can be rescued by manipulations of the retinoid content of the mother’s diet, which demonstrates that the phenotypes observed are related to the known activities of the two enzymes [60,222]. The roles of RDH10 and DHRS3 are non-redundant during development and are conserved in other vertebrate species examined ([223,224]. Both RDH10 and DHRS3 are expressed in a wide variety of tissues in postnatal life, however, the contribution of RDH10 and DHRS3 to vitamin A homeostasis outside development is not known. There is evidence for a role of RDH10 in postnatal vitamin A metabolism. For example, RDH10 was shown to be required for spermatogenesis and hemizygous *Rdh10*^+/−^ have slightly decreased levels of RA and increased adiposity [64,66]. Genetic studies have implicated other SDR enzymes with retinoid oxidoreductase activity in vitamin A metabolism in adult tissue such as skin (RDHE2 and RDHE2S), liver (RDH11), testes (RDH11), fat (RDH1), and in the visual system (RDH5, RDH8 and RDH12) [92,225,226,227,228].

The conversion of retinol to retinaldehyde is subject to control by RA. The expression of *Dhrs3* is consistently upregulated in models of RA excess [229,230]. Though data suggests that *Dhrs3* is a direct target of RAR, no functional RARE has so far been demonstrated. Meanwhile, the expression of *Rdh10* is suppressed in the presence of RA excess [59]. In addition, RDH10 and DHRS3 also influence each other at protein level. A significant number of SDRs are present as multimers, mostly homodimers and homotetramers [231]. DHRS3 and RDH10 proteins share 40% sequence identity which raises the possibility that they also interact with one another. Indeed, studies by Adams et al. show that RDH10 and DHRS3 not only form homo-oligomers but also DHRS3-RDH10 hetero-oligomers [215,232,233]. These interactions were observed in the case of RDH10 and DHRS3 overexpressed in cells, but there is evidence that this association persists in the case of endogenous proteins. The model emerging from these studies suggests that association of RDH10 with DHR3 forms a bifunctional retinoid oxidoreductive complex (ROC), which through reciprocal interactions stabilizes and increases the activity of component proteins. By catalyzing antagonistic reactions, the ROC ensures RA homeostasis despite fluctuations in the starting level of retinol precursor. The ROC complex is composed of type I integral ER-resident membrane proteins oriented towards the cytoplasm [233]. Structural modeling studies suggest that the membrane dynamics may influence the heteromeric composition of the ROC; however, more work is needed to untangle the mechanisms by which ROC controls the formation of RA [233].

The second step in the conversion of retinol to RA is the irreversible oxidation of retinaldehyde to RA which is mediated by cytosolic retinaldehyde dehydrogenase 1, 2 or 3 (RALDH1-3 encoded by *Aldh1a1-3*) enzymes. Of the three, RALDH2 is critical throughout development and is responsible for RA synthetic capacity of some adult tissues such as hematopoietic and reproductive tissues [55,234,235,236]. RALDH1 and RALDH3 have more restricted expression pattern and are important for RA synthesis in tissues such as bone, fat, and developing eye and nasal regions [65,237,238,239,240,241,242]. High levels of RA lead to reduced expression of *Raldh1* and *2* [59]. There is evidence that suppression of the expression of *Raldh1* by RA is mediated by direct RAR binding and through interactions with GADD153-C/EBP-beta [243,244]. In addition to RALDH enzymes, there is evidence that the molybdo-flavoenzyme aldehyde oxidase (AOX) contributes to RA synthesis in vivo [245,246]. The cytochrome P450 enzyme CYP1B1 can also contribute to the formation of RA [247,248,249]. However, both AOX and CYP1B1 can oxidize a wider range of endogenous and exogenous substrates in addition to retinoids.

## 9. Cellular Fate of RA and RA Breakdown

Newly formed RA is available for signaling via RAR within the same cell (cell autonomously) or it can be secreted to signal to neighboring cells. Cell autonomous RA-signaling can contribute to the feedback control mechanism that regulates vitamin A metabolism. Paracrine RA-signaling from a RA-source cell to an RA-responder cell is important for the morphogen functions of RA. RA patterns development through both gradients of decreasing RA as well as through fields of RAR-signaling. RA gradients require not only a source of RA but also a catabolic sink. On the other hand, fields of RAR signaling need to be broken up by zones where RAR-signaling is extinguished [250,251,252]. Though extracellular RA was shown to bind some non-specific plasma proteins [200], very little is known regarding how RA moves from cell to cell and whether the intercellular movement of RA is regulated by or require any cellular factors. Within cells, RA is bound to high affinity cellular RA binding proteins 1 and 2 (CRABP1, 2) which play an important role in channeling RA towards its alternate fates of signaling or degradation (refs. [91,253,254,255] reviewed in [94,256]). Both *Crabp* genes respond to RA either directly (Crabp2) or indirectly (*Crabp1*) [108,257].

RA binding to RAR leads to RAR-RXR receptor activation. Interestingly, all three genes coding for RA receptors are induced by RA creating a feedforward loop which, in theory, could serve to coordinate the timing of ligand synthesis with RAR expression [72,258,259,260,261,262,263]. The termination of RAR-signaling is a poorly understood event, however, there is evidence that RA binding induces ubiquitin-mediated degradation of RAR via the proteasome [264,265,266,267].

RA oxidation involves hydroxylation of the C4 or C18 positions of the ionone ring and is catalyzed by cytochrome p450 enzymes of the CYP26 family, namely CYP26A1, B1, or C1. The expression of *Cyp26a1-c1* displays developmental and tissue specificity, while the CYP26 enzymes exhibit distinct preference with regard to their retinoid substrate [91,268,269]. For example, CYP26A1 and B1 are responsible for the initial oxidation to produce 4-hydroxy-RA while CYP26C1 is more efficient in clearing 4-oxo-RA [255]. Studies support transcriptional activities for ring oxidized retinoids in certain adult tissues such as skin, and in *Xenopus* development [23,270,271]. However, oxidized-RA metabolites do not seem to contribute to the developmental functions of vitamin A in mouse [53]. There is evidence that other families of P450 enzymes including CYP2 and CYP3 families could also contribute to RA oxidation in some settings [272,273,274].

A mitochondrial adrenodoxin-coupled P450 enzyme, CYP27C1 is involved in the desaturation of the 3–4 double bond of the ionone ring of retinoids. This activity leads to formation of 3,4-didehydroretinoids [275]. Such 3,4-didehydroretinoids include vitamin A2 (all-*trans*-3,4-didehydroretinol) which is found in human skin [276], and is also an important visual chromophore of freshwater fish and amphibians [277,278].

Genetic and pharmacologic approaches confirm that the CYP26 family enzymes act as the primary contributor to RA degradation in vivo [279,280,281,282,283]. To guard against excess RA, the expression of *Cyp26a1* is induced by RA which serves to restore appropriate RA levels. This is part of an important regulatory negative feedback loop where RA induces its own degradation [284,285,286,287,288,289]. HNF4A cooperates with RAR in the regulation of *Cyp26a1* [284,290,291,292]. However, developmentally, CYP26 enzymes play even more complex roles in RA metabolism. In conjunction with their task of monitoring RA metabolism, f CYP26 enzymes play a role in establishing RA gradients as well as RA-free zones which are required for RA-mediated developmental processes [250,252].

Phase II metabolism and clearance of RA involves its conjugation via various glucuronosyltransferases which impart oxidized-RA metabolites with a higher aqueous solubility [293,294,295,296]. In addition to glucuronides of RA there is evidence that retinol can also be glucuronidated [297]. Microbiome expressed glucuronidases play an important role in the reactivation and enterohepatic recirculation of conjugated drugs and hormones including isotretinoin (13-*cis*-RA) [298]; however, it is not clear if the microbiome plays any role in the reactivation of retinoyl glucuronide (RAG) derived from endogenous RA under physiological circumstances.

## 10. Homeostasis in Vitamin A Metabolism

Vitamin A metabolism can be affected by both genetic and environmental influences. Despite the wide range in dietary vitamin levels and format of vitamin A precursors (preformed retinol, retinyl esters and provitamin A carotenoids) organisms are ordinarily able to achieve a relatively stable level of serum retinol. A stable level of precursor allows target cells to derive visual chromophore, and a context-appropriate level of RA to sustain its transcriptional functions. Analysis of the effect of RA treatment or VAD diet on the expression of various retinoid genes provides a picture of adaptive responses which preserve vitamin A homeostasis (summarized in Table 1 and depicted in Figure 3), but the evidence is still incomplete and only available for specific tissues. Additionally, listed responses describe transcript level, and will need to be confirmed at protein or protein activity level. Despite these limitations enough is known to form some preliminary conclusions. First, adaptations to excess RA involve most retinoid biotransformations and overlap with pathways that play a role in shaping the morphogenetic roles of RA involving:

1. upregulation of genes responsible for sequestering RA precursors such as *Crbp1* and *Lrat*.
2. upregulation of genes responsible for opposing RA formation (*Dhrs3*) and the degradation of RA (*Cyp26a1*)
3. downregulation of genes involved in the synthesis of RA (*Rdh10*, *Raldh2*)
4. downregulation of genes involved in the uptake of carotenoids (*Srb1*) and conversion of β-carotene to retinaldehyde (*Bco1*)

The second observation is more challenging. Though retinoid genes seem to respond to a VAD diet, a clear pattern is not apparent, and, thus far, there is no evidence of an orchestrated response to augment vitamin A absorption or decrease its catabolism in a state of VAD. This indeterminate response could simply be a limitation imposed by the currently available data. Hopefully, more thorough analyses comparing the expression of retinoid genes from different tissues of VAD animals could shed more light on how an organism responds to VAD to promote absorption and/or mobilization of retinol from stores for utilization by target tissues.

Even more challenging is the interpretation of the functional significance of the regulation of RBP4 receptors by RA. Evidently, *Stra6* is upregulated by RA. Given its role in bidirectional transport of retinol, it is tempting to speculate that upregulation of STRA6 by RA serves to counter systemic retinoid excess. Thereby induction of *Stra6* in target tissues would cause target tissues to take up excess retinol from serum. Could this response potentially cause cytotoxic effects in target tissues? Alternatively, it is possible that STRA6 only responds to local excess of cellular retinol. In this case local upregulation of *Stra6* causes the export of retinol from cells to serum apo-RBP4 to mitigate cellular excess. Equally puzzling is the observed negative correlation between *Rbpr2* expression and retinoid status. Downregulation of *Rbpr2* in response to RA is not coherent with the logic that liver would serve to absorb and clear excess retinol to avoid toxicity. Clearly, more work is needed to understand the biological impact and meaning of the regulation of RBP4 receptors by RA. Given that the RARE responsible for the regulation of the expression of *Stra6* has now been identified, there is an opportunity to interrogate the functional significance of the regulation of *Stra6* by RA via genetic approaches.

Many retinoid genes have been shown to be upregulated or downregulated in response to RA, but we seldomly know if the regulation by RAR is direct or indirect. This may seem to be a trivial aspect, but it has important implications for the dynamics and impact of RA feedback regulation. Genes that are indirectly regulated by RAR require an intermediate transcription factor which itself is directly or indirectly regulated by RAR (Figure 1). ISX is such an RA-induced transcription factor, which orchestrates and integrated network in provitamin A carotenoid metabolism by suppressing the expression of *Bco1* and *Srb1* [95]. Indeed, genetic ablation of ISX leads to dramatic increases in provitamin A carotenoid absorption and conversion. Even so, SR-B1 is also involved in the uptake of other vitamins and lipids like lutein, tocopherol and vitamin K [299,300,301], so, in theory, there is potential that high levels of dietary preformed vitamin A could cause decreased uptake of unrelated lipids.

For many genes known to be *directly* regulated by RAR (based on transcription dynamics, effect of translation inhibitors) a functional RARE has yet to be identified. In silico analysis of the genomic sequence of RA-responsive genes have uncovered sequences with similarity to DR response elements which are typically bound by RAR [302]. However, predictions based on sequence alone often fail to identify with any degree of certainty a RARE involved in the regulation of a specific gene. Even having identified an RAR-bound site in the vicinity of an RA-controlled gene does not guarantee that the particular RARE is responsible for the effects of RA on the expression of the neighboring gene. At the individual gene level, the functional relevance of a response element can be investigated via in vitro DNA-binding, mutagenesis, and by examining the activity of reporters driven by minimal promoters incorporating the putative RAR binding region. Genome editing/mutagenesis of the putative RARE can provide conclusive proof that the identified element functions as a genuine RARE in the native chromatin context [81,203,303]. Genome-wide approaches based on mapping chromatin interactions and enhancers could serve to bridge this gap in the future [304,305]. In such an example, an approach based on chromosome conformation capture combined with sequencing (3C-Seq) was used to analyze the enhancer-gene relationships that shape the RXR-mediated regulation of macrophages [306]. Studies of the regulation of RA metabolism usually tackle one factor at a time which makes it difficult to have a coherent picture of this broad regulatory network. Studies by Parihar et al. have examined the dynamic transcriptomics of *Xenopus* embryos exposed to RA or to inhibitors of RA synthesis [307]. The study elegantly illustrated the robustness of thenetwork that regulates retinoid homeostasis, and provides evidence that the equilibrium that keeps RA within a narrow range of normal is derived from a dynamic correcting oscillatory behavior.

The regulatory mechanisms that govern vitamin A metabolism usually demonstrate robustness and resilience but can sometimes be hyperactive evoking maladaptive responses seen in cases of hormone withdrawal. Several studies of the immediate and late effects of RA on mouse fetal development have painted a fascinatingpicture of the capacity of the vitamin A regulatory feedback mechanism. For example, pharmacological doses of RA result at first in vast excess of RA in target tissues, but at later timepoints the same RA insult causes a paradoxical deficiency [308]. Moreover, some of the developmental defects elicited by RA treatment were prevented by a subsequent dose of RA which mitigated the RA deficiency that follows initial excess. Overcompensation was also observed following genetic manipulation of RA metabolic enzymes and RAR receptors [309,310,311]. Hundreds of studies of RA toxicity and teratogenicity have been conducted over the years on the premise that the effects observed are a result of RA excess, when in fact, some of the effects of RA treatment may very well reflect the ensuing deficiency of endogenous RA.

In conclusion, there is clear evidence for powerful feedback mechanisms that operate via RA–RAR/RXR and which act on retinoid enzymes, binding proteins, and transporters. The molecular mechanisms of RA feedback regulation are starting to emerge for some pathways such as ISX [93]. At the same time, as seen in studies using time-series transcriptomics in tractable models such as *Xenopus* [307], feedback regulation is both dynamic and complex. Future directions in this research could involve both exploring the molecular mechanisms of vitamin A homeostasis and seeking to gain more insight in the inter-organ dialogue required to maintain vitamin A homeostasis.

## Figures and Tables

**Table 1:** Regulation of retinoid genes in response to RA Treatment and VAD Diet. Only retinoid genes currently known to respond to RA are listed.

Role in Vitamin A Metabolism	Gene Name	Acronym	Effect of VAD on Gene Expression	Effect of RA on Gene Expression
Signaling	Retinoic acid receptors	RARα RARβ RARγ	Downregulated in some tissues of VAD rats and quail [312,313]	Directly upregulated in response to RA via conserved RARE [72,258,259,260,261,262]
	Retinoid X receptors	RXRα RXRβ RXRγ	Downregulated of *Rxra* and *Rxrb* in hearts of VAD rats, corrected with VA supplementation. [314]	Not clear if *Rxr* genes are RAR-targets
Conversion of provitamin A carotenoids to retinol	B-carotene-15,15-dioxygenase 1	BCO1	Upregulated in VAD mice [130].	Expression is suppressed by RA via RAR-mediated induction of the transcription factor ISX [115,128,129,130,131,132]
Storage	Lecithin retinal acyltransferase	LRAT	Protein and transcript levels of LRAT decrease in the many tissues of VAD animals [315,316,317,318,319]. There is evidence that the magnitude and direction of response is tissue-specific.	Indirectly upregulated in response to RA, suggested by fact that upregulation pf LRAT and LRAT activity by RA is blocked by the translation inhibitor, cycloheximide [163,316,320,321]. No functional RARE sites have been identified. A genomic region of the *Lrat* promoter confers RA-inducibility and contains binding sites for SP1 [109] and GATA transcription factors [107]
Retinol Binding Proteins	Cellular retinol-binding proteins	CRBP1	Decreased expression of *Rbp1* in VAD rats [322,323,324]	Upregulated by RAR via a direct mechanism unaffected by cycloheximide and including a functional RARE [108,325,326]
		CRBP2	Upregulated in the intestine of VAD rats [324]	Not clear if regulated in response to RA. Promoter appears to harbor a poorly conserved response element for RXR or HNF-4 [327,328] and whose physiological relevance is currently, unclear [105,329].
	Retinol binding protein	RBP4	VAD causes reduced secretion of RBP4 from liver cells [155,200,201]	Expression induced in response to RA [197,198] but has not been clearly demonstrated to be via direct mechanism or to harbor a functional RARE.
RBP4 Receptors	Stimulated by retinoic acid 6	STRA6	VAD causes expansion of domains of expression of *STRA6* in quail embryos [330]. Alternatively spliced *Stra6* mouse isoforms are differentially regulated by VAD [203].	Directly induced by RA via a functional RARE [173,174,202,203]
	Retinol binding protein receptor 2	RBPR2	Expression is inversely correlated with liver retinol stores [180].	Expression is downregulated by RA or retinol treatment [180].
RA synthetic enzymes	Retinol dehydrogenase 10	RDH10	Expression of *Rdh10* is upregulated in genetic models of RA-deficiency [217]	*Rdh10* is negatively regulated by RA [224,331]. Β-carotene supplementation leads to downregulation of Rdh10 [332]. *Rdh10* is downregulated in genetic models of RA-excess [59]
	Retinaldehyde dehydrogenases 1-2	RALDH1-2	VAD causes upregulation of *Raldh1* and downregulation of *Raldh*2 in rat testes [333],	*Raldh1* and *Raldh*2 are downregulated in genetic models of RA-excess in mouse [59]. Suppression of *Raldh1* expression by RA is via direct RAR binding [243,244]
Enzymes which prevent RA formation or reduce RA levels	Short-chain dehydrogenase reductase family member 3	DHRS3	Expression is decreased in the liver and hearts of VAD rats [63,229].	Directly upregulated by RA, though a functional RARE has not been identified [229,230].
	Cytochrome P450 26 A1	CYP26A1	CYP26A1 is downregulated in liver and pancreatic tissues of VAD mice [334,335].	Directly upregulated via an identified RARE [288,289]. HNF4A cooperates with RAR in the regulation of CYP26A1 [284,290]
	Cytochrome P450 enzymes family 2 C22	CYP2C22		Directly upregulated by RA [272]
RA binding proteins	Cellular retinoic acid-binding proteins	CRABP1 CRABP2	*Crabp1* and *Crabp2* are downregulated in liver and pancreatic tissues of VAD mice [334]	*Crabp1* is indirectly upregulated by RA [108]. *Crabp2* is directly upregulated by RA via an identified RARE [257].

## Abbreviations

AKR	aldo-keto reductase
CRBP	cellular retinol binding protein
CRABP	cellular retinoic acid binding protein
CYP	cytochrome P450
DHRS	dehydrogenase/reductase (SDR family) member
LRAT	lecithin:retinol acyltransferase
NAD	nicotinamide adenine dinucleotide
NADP	nicotinamide adenine dinucleotide phosphate
NHR	nuclear hormone receptor
RA	all-*trans*-retinoic acid
RAR	retinoic acid receptor
RXR	retinoid X receptor
RALDH	retinaldehyde dehydrogenase
RDH	retinol dehydrogenase
SDR	short-chain dehydrogenases reductase
TTR	transthyretin
